# Abatacept, Golimumab, and Sarilumab as Selected Bio-Originator Disease-Modifying Antirheumatic Drugs with Diverse Mechanisms of Action in Their Current Use in Treatment

**DOI:** 10.3390/jcm14062107

**Published:** 2025-03-19

**Authors:** Piotr Kawczak, Igor Jarosław Feszak, Tomasz Bączek

**Affiliations:** 1 Department of Pharmaceutical Chemistry, Faculty of Pharmacy, Medical University of Gdańsk, 80-416 Gdańsk, Poland; tomasz.baczek@gumed.edu.pl; 2 Institute of Health Sciences, Pomeranian University in Słupsk, 76-200 Słupsk, Poland; igorfeszak@gmail.com; 3 Department of Nursing and Medical Rescue, Institute of Health Sciences, Pomeranian University in Słupsk, 76-200 Słupsk, Poland

**Keywords:** inflammatory arthritis, targeted treatment, boDMARDs, costimulatory blockers, TNFi, IL-6Ri

## Abstract

**Background/Objectives**: Arthritis encompasses a range of joint-related conditions, including osteoarthritis and rheumatoid arthritis, along with inflammatory diseases such as gout and lupus. This research study explores the underlying causes, challenges, and treatment options for arthritis, aiming to enhance the effectiveness of therapies. **Methods**: This research study evaluated current treatment strategies and examined the effectiveness of selected biological disease-modifying antirheumatic drugs (bDMARDs), i.e., abatacept, golimumab, and sarilumab, with a focus on emerging drug classes and their distinct mechanisms of action. **Results**: Biologic DMARDs like abatacept, golimumab, and sarilumab offer hopeful treatment alternatives for patients who fail to respond to conventional therapies. However, individual outcomes differ because of the disease’s complexity and the influence of accompanying health conditions. **Conclusions**: Treating arthritis continues to be challenging due to its numerous underlying causes and the varied ways in which patients respond to treatment. Although biologics and targeted therapies have brought progress, additional research is needed to identify new treatment targets and enhance patient results.

## 1. Introduction

Arthritis is a joint disorder, with common types like osteoarthritis and autoimmune rheumatoid arthritis, along with inflammatory forms such as gout, lupus, and septic arthritis [1]. Rheumatoid arthritis (RA) is a chronic, systemic autoimmune disease primarily characterized by joint inflammation and damage, where autoantibodies and chronic inflammation target various molecules and altered self-epitopes [2,3]. It affects roughly 1% of the global population, especially individuals aged 20–40 and those over 75, with women being more susceptible. RA’s impact extends beyond joint destruction to include systemic effects—such as cardiovascular, respiratory, and neurological complications—and it is often accompanied by comorbidities like diabetes, heart disease, and COPD, which contribute to increased mortality [4,5,6].

Despite a broad array of treatments—including analgesics, glucocorticoids, and biologic response modifiers—some patients have suboptimal outcomes due to the disease’s complex mechanisms, including dysregulated programmed cell death [7]. Key immune cells, such as memory B cells and invariant NKT cells, contribute to RA’s onset, and the variable clinical response to treatments like DMARDs, glucocorticoids, NSAIDs, and cytokine inhibitors underscores the need for a deeper understanding of the disease’s cellular and molecular basis [8,9,10].

In recent years, non-conventional synthetic DMARDs (ncs-DMARDs), including biological DMARDs (bDMARDs) and targeted DMARDs (tDMARDs), have been used for rheumatoid arthritis (RA) patients who do not respond well to or cannot tolerate conventional synthetic DMARDs (csDMARDs). With the emergence of new treatments like kinase inhibitors and biosimilars, a revised classification system for DMARDs has been introduced. The new system distinguishes biological DMARDs into original (boDMARDs) and biosimilars (bsDMARDs), including drugs like abatacept, adalimumab, and newer options, such as clazakizumab and ixekizumab. Synthetic DMARDs are now categorized into conventional (csDMARDs) and targeted synthetic (tsDMARDs), with tsDMARDs including drugs like tofacitinib and baricitinib, along with others used for different conditions, such as imatinib. csDMARDs include methotrexate, sulfasalazine, leflunomide, hydroxychloroquine, and gold salts [11,12].

In order to understand the criteria for including individual drugs in rheumatological diseases, we will use information from the latest European Alliance of Associations for Rheumatology (EULAR) and American College of Rheumatology (ACR) guidelines for rheumatoid arthritis (RA) from the year 2022 (Table 1) [13].

A score < 6 means that the patient does not meet the criteria for definite RA but may be reassessed over time. A score ≥ 6 means that the patient is classified as having definite RA, provided no other condition better explains the symptoms [13].

The management of rheumatoid arthritis (RA) follows a structured, evidence-based strategy that emphasizes early detection, prompt treatment, and continuous monitoring. Diagnosis is based on ACR/EULAR criteria, which consider joint involvement, serological markers such as rheumatoid factor (RF) and anti-citrullinated protein antibodies (ACPA), inflammatory markers like C-reactive protein (CRP) and erythrocyte sedimentation rate (ESR), and symptoms persisting for more than six weeks. Disease activity should be assessed by using standardized tools. Methotrexate (MTX) is the preferred initial treatment, often paired with short-term glucocorticoids for quick symptom relief, though these should be tapered off as soon as possible. If MTX is not an option, alternatives include leflunomide and sulfasalazine. Regular monitoring every 1–3 months helps guide treatment adjustments. If there is no noticeable improvement after three months or the treatment goal is not met within six months, therapy should be modified based on prognostic factors. For patients without high-risk indicators, switching to another csDMARD may be sufficient. However, in cases of severe disease, early joint damage, or high autoantibody levels, adding a bDMARD or tsDMARD is recommended. JAK inhibitors should be prescribed with caution due to their potential cardiovascular and cancer-related risks. If the first bDMARD or tsDMARD does not work, switching to a different drug class is advised. Once remission is maintained for at least six months, a gradual reduction in DMARD dosage may be considered, but complete discontinuation is not recommended to prevent relapse [14]. We took the liberty of presenting a brief summary of above process in Table 2.

Disease activity should be tracked by using tools like the “Disease Activity Score assessing 28 joints” (DAS28) or the “Clinical Disease Activity Index” (CDAI) [15,16]. The DAS28 is calculated by using a combination of clinical and laboratory parameters. The total score ranges from 0 to 9.4 and is derived from the following components [15,16]:

1. Tender Joint Count (TJC)—number of tender joints out of 28 assessed joints.
2. Swollen Joint Count (SJC)—number of swollen joints out of 28 assessed joints.
3. ESR or CRP.
4. Patient Global Assessment (PGA)—patient’s self-reported assessment of disease activity, rated on a Visual Analog Scale (VAS) from 0 to 100 mm.

The formula is DAS28 = 0.56 × √(TJC) + 0.28 × √(SJC) + 0.70 × ln(ESR) + 0.014 × GH [15].

We presented the interpretation of the final DAS28 score in the form of Table 3.

The CDAI is a simpler tool that does not include laboratory values, making it easier to use in clinical practice. The total score ranges from 0 to 76 and is calculated as follows [15,16]:

1. TJC—number of tender joints out of 28 assessed joints.
2. SJC—number of swollen joints out of 28 assessed joints.
3. PGA—on a 0–10 cm (0–100 mm) Visual Analog Scale.
4. Evaluator Global Assessment (EGA)—physician’s assessment of disease activity on a 0–10 cm VAS;

The formula is CDAI = TJC + SJC + PGA + EGA [15].

We presented the interpretation of the final CDAI score in the form of Table 4.

Key differences between the DAS28 and the CDAI include ESR or CRP, making the former dependent on laboratory tests. The CDAI relies only on clinical examination and patient-reported assessments, making it a practical tool for rapid decision making in clinical settings. Both scores help determine disease severity, guide treatment escalation, and monitor remission in rheumatoid arthritis patients [15,16].

## 2. Bio-Originator Disease-Modifying Antirheumatic Drugs (boDMARDs)

Approximately 30–50% of patients do not achieve a sufficient response to conventional DMARDs. Biologic DMARDs should be considered if treatment with methotrexate, either alone or in combination with other traditional DMARDs, fails to produce adequate results after 2–6 months. All currently available biologic DMARDs are antibody-based therapies designed to target key inflammatory or immune pathways and are administered via infusion or subcutaneous injection. The introduction and use of biologic DMARDs have significantly transformed the management of rheumatoid arthritis (RA) [17].

The selection of biologic disease-modifying antirheumatic drugs (bDMARDs) is guided by multiple factors, including patient-specific characteristics, comorbidities, and prior treatment experiences. For individuals with elevated cardiovascular risk, abatacept may be the preferred choice, as research suggests that it is linked to a lower likelihood of cardiovascular events compared with TNF inhibitors like golimumab [18]. Patients with a history of malignancy may also opt for abatacept over TNF inhibitors, as they have demonstrated a more favorable safety profile in this regard [19]. When patients do not respond to an initial biologic therapy, switching to a drug with a different mechanism of action—such as transitioning from a TNF inhibitor to an IL-6 inhibitor like sarilumab—tends to be more effective than remaining within the same drug class [20].

Long-term safety considerations play a crucial role in choosing the appropriate biologic therapy. Although all bDMARDs carry an increased risk of infections, golimumab and sarilumab may pose a higher likelihood of bacterial and viral infections, including tuberculosis reactivation [21]. While long-term studies do not suggest a significant rise in cancer risk among bDMARD users, data on TNF inhibitors remain inconclusive [19]. In terms of cardiovascular safety, abatacept appears to have a more favorable profile compared with TNF inhibitors, making it a preferred option for patients with pre-existing cardiovascular conditions [21].

The use of biomarkers to predict treatment response is gaining importance in personalized medicine. For instance, elevated rheumatoid factor (RF) and anti-CCP antibody levels are linked to a better initial response to abatacept, which often results in a reduction in RF levels, while anti-CCP levels typically remain unchanged [22]. Increased levels of C-reactive protein (CRP) and interleukin-6 (IL-6) may indicate a higher likelihood of response to sarilumab, as its mechanism of action reduces these inflammatory markers [21]. Additionally, research is ongoing to explore the influence of genetic polymorphisms on golimumab response, though further studies are needed in this area [20].

### 2.1. Co-Stimulation Inhibitor (CTLA4-Ig)—Abatacept

**Figure 1:**
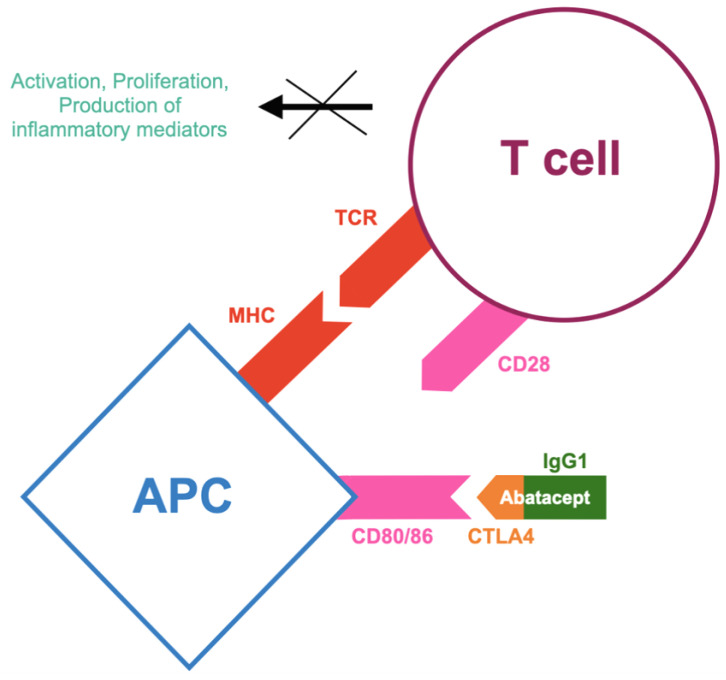
Mode of action of abatacept according to [24], where APC—antigen-presenting cells; MHC—major histocompatibility complex; TCR—receptor on the T cell; CTLA4—human cytotoxic T lymphocyte-associated antigen 4; IgG1—genetically engineered fragment of the Fc region of human immunoglobulin G1; CD(28/80/86)—clusters of differentiation. Abatacept functions by utilizing its extracellular CTLA-4 domain to bind to CD80/CD86 receptors. This interaction prevents or disrupts the binding of these receptors to CD28. By selectively inhibiting this connection, abatacept effectively blocks the crucial second signal required for immune activation. As a result, T-cell activation is suppressed, modulating the immune response.

Abatacept is an effective treatment for various autoinflammatory diseases, particularly rheumatoid arthritis (RA). Its primary mechanism involves inhibiting T-cell activation by blocking CD28 signaling, but it also influences regulatory T cells, monocytes, macrophages, osteoclasts, and B cells [23]. A fully humanized fusion protein, abatacept combines the extracellular domain of CTLA-4 with a modified IgG1 Fc fragment to prevent CD80/CD86 from binding to CD28, thereby reducing inflammatory responses. It is FDA-approved for RA, juvenile idiopathic arthritis (JIA), and active psoriatic arthritis (PsA) [24,25,26,27]. CTLA-4 is a critical negative regulator of T-cell activation, competing with CD28 for B7 ligands. Its absence can lead to autoimmune diseases, highlighting its role in immune tolerance [28]. Abatacept (Orencia), produced by using recombinant DNA technology, was initially available as an intravenous (IV) infusion before the introduction of a subcutaneous (SC) version. T-cell activation, essential in RA, requires both antigen recognition and co-stimulation. By binding CD80/CD86, abatacept reduces CD28 signaling, limiting T-cell activation and cytokine production [29,30]. The mechanism of action of abatacept is presented in Figure 1.

As a biologic disease-modifying antirheumatic drug, abatacept is effective as monotherapy or combined with traditional DMARDs, particularly in early-stage RA and patients with high anti-citrullinated protein antibody (ACPA) levels. Early intervention with abatacept slows disease progression and maintains efficacy post-treatment without new safety concerns [31]. Available as a monthly IV infusion or weekly SC injection, abatacept alleviates RA symptoms, slows structural damage, and improves physical function, even in patients unresponsive to methotrexate or TNF inhibitors. Transitioning between IV and SC forms does not affect efficacy or safety, and abatacept is well tolerated in older adults [32,33]. In juvenile idiopathic arthritis (JIA), abatacept rapidly and durably reduces inflammation with a strong safety profile. Studies suggest similar efficacy and safety compared to adalimumab and etanercept [34,35]. It may also improve ACR70 response rates compared with tocilizumab, demonstrating superior clinical outcomes in RA with fewer adverse events than other biologic DMARDs [36,37,38]. Abatacept is approved for PsA in patients unresponsive to conventional DMARDs but is not recommended for those with uncontrolled skin lesions or axial involvement [39]. It has shown promise in treating giant-cell arteritis (GCA), chronic graft-versus-host disease (cGVHD), and acute GVHD prevention in hematopoietic cell transplantation, particularly in HLA-mismatched transplants [40,41,42,43,44]. Although abatacept failed to meet primary endpoints in systemic lupus erythematosus (SLE) trials, its immune-modulating properties suggest potential for lupus nephritis treatment [45]. It may also be effective for refractory myositis, and further trials are needed [46]. In type 1 diabetes, abatacept did not prevent glucose intolerance but helped maintain insulin secretion, indicating potential for immune modulation in beta-cell preservation [47,48]. A rare side effect associated with abatacept is panniculitis, though its histological pattern remains unclear, warranting further research [49]. Other severe adverse effects, including opportunistic infections and lymphoproliferative disorders, have also been observed [50,51].

**Figure 2:**
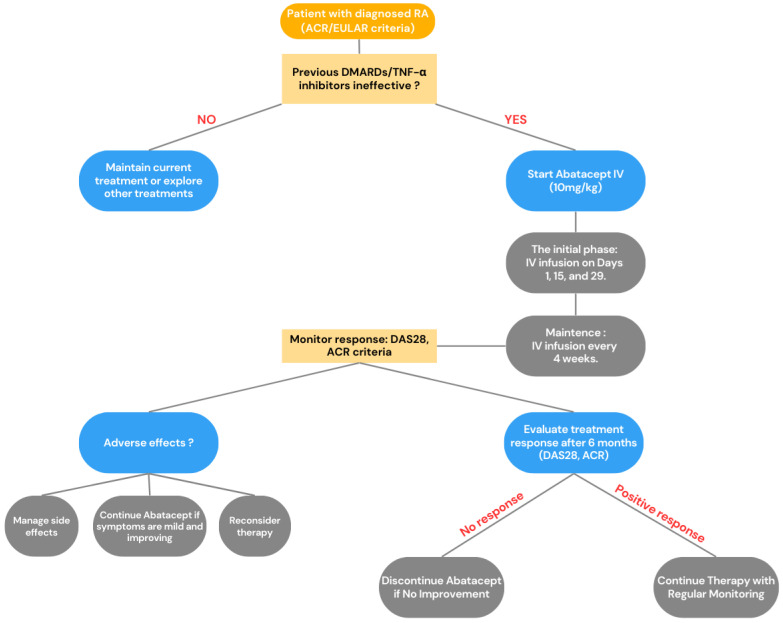
Brief summary of abatacept therapy pathway for rheumatoid arthritis (RA) according to [52], where RA—rheumatoid arthritis; ACR—American College of Rheumatology; EULAR—European League Against Rheumatism; DMARDs—disease-modifying antirheumatic drugs; TNF-α—tumor necrosis factor alpha; DAS28—Disease Activity Score-28.

Abatacept is prescribed for individuals with active, moderate-to-severe rheumatoid arthritis who have not responded adequately to previous treatment with disease-modifying antirheumatic drugs or TNF-α inhibitors. To qualify for this therapy, patients must meet the ACR/EULAR criteria for RA and continue to show signs of disease activity despite standard treatment. The medication is administered intravenously at a dose of 10 mg/kg, starting with infusions on days 1, 15, and 29, followed by ongoing maintenance infusions every four weeks. Each infusion takes approximately 30 min to complete [13,31,32,52]. Abatacept is typically used alongside other antirheumatic drugs, with methotrexate (MTX) being the preferred option. Depending on the patient’s specific needs, other DMARDs, such as sulfasalazine or leflunomide, may also be considered. Additionally, stable treatment regimens may include nonsteroidal anti-inflammatory drugs (NSAIDs) and low-dose glucocorticoids (≤10 mg/day of prednisone or an equivalent) [52]. Disease activity is routinely monitored by using the DAS28 score, while treatment response is assessed based on ACR improvement criteria [31,52]. Safety monitoring is essential, particularly for potential infections like tuberculosis or upper respiratory tract infections. Regular laboratory tests, including blood counts, liver function assessments, and C-reactive protein (CRP) levels, are conducted throughout treatment. Abatacept is contraindicated in patients with active or chronic infections, such as tuberculosis or hepatitis B/C, and should not be combined with other biologic agents, especially TNF-α inhibitors, due to increased infection risk. Special caution is necessary in individuals with comorbidities such as chronic lung disease, diabetes, or heart failure. If no meaningful improvement, such as achieving an ACR20 response (Table 5), is observed within six months, discontinuing treatment should be considered [13,31,52]. For patients who respond well, therapy continues with regular evaluations to ensure effectiveness and tolerability. Abatacept administration follows a structured infusion protocol, requiring ongoing assessment of its benefits and risks, particularly regarding infection susceptibility and potential interactions with other biologic treatments. To visualize the decision-making algorithm for the use of abatacept in RA treatment, we created a graphical summary in Figure 2.

The ACR improvement criteria quantify the percentage reduction in RA symptoms from baseline following treatment [13,31]. This evaluation includes both clinical and laboratory measures, such as (1) Tender Joint Count (TJC), (2) Swollen Joint Count (SJC), (3) patient’s pain assessment using a Visual Analog Scale (VAS), (4) Patient’s overall assessment of disease activity, (5) physician’s overall assessment of disease activity, (6) functional disability measured by the Health Assessment Questionnaire (HAQ), and (7) Inflammatory markers, including erythrocyte sedimentation rate (ESR) or C-reactive protein (CRP) [13,31,52]. The interpretation of these criteria is as follows: ACR20 requires at least a 20% improvement in both TJC and SJC, along with a 20% improvement in at least three of the remaining five indicators. ACR50 and ACR70 follow the same pattern, requiring 50% and 70% improvements, respectively. ACR20 is generally regarded as the minimum threshold for clinical efficacy in research studies, while ACR50 and ACR70 indicate greater symptom relief and stronger therapeutic effectiveness [13]. Higher ACR scores reflect a more substantial response to treatment, suggesting better disease management and potentially slower progression. These criteria are fundamental tools in both clinical trials and everyday practice for objectively assessing and comparing treatment outcomes.

### 2.2. TNF Inhibitor—Golimumab

In the mid-1980s, researchers introduced the concept of using targeted antibodies to block tumor necrosis factor (TNF) and restore cytokine balance. This led to the development of infliximab, a chimeric monoclonal antibody designed to neutralize TNF while sparing lymphotoxin (TNF-β). Following this breakthrough, additional biologic agents, such as etanercept, adalimumab, certolizumab, and golimumab, were developed, each with distinct structural and pharmacodynamic properties. These TNF-α inhibitors have been effective in managing rheumatoid arthritis (RA) by reducing disease activity, slowing joint damage, and preserving function. Intravenous (IV) golimumab has shown similar efficacy in controlling disease progression while maintaining a favorable safety profile. Notably, IV golimumab is the only fully human TNF-α inhibitor administered intravenously in a short 30 min infusion, though its long-term adoption remains uncertain [53,54,55,56,57,58]. Anti-TNF-α therapies are used to treat various rheumatologic and inflammatory conditions, including RA, ankylosing spondylitis (AS), psoriatic arthritis (PsA), and inflammatory bowel diseases such as Crohn’s disease and ulcerative colitis. Additionally, they are utilized in dermatology for plaque psoriasis. Five anti-TNF-α agents are available, infliximab, etanercept, adalimumab, certolizumab, and golimumab, with biosimilars for the first three. Golimumab, a human IgG1κ monoclonal antibody, neutralizes TNF-α by preventing its interaction with receptors, thereby reducing inflammation. While infliximab, adalimumab, and golimumab can bind complement and Fc receptors, certolizumab features a modified hinge region with polyethylene glycol chains to enhance solubility and extend half-life [59,60,61,62,63,64,65].

**Figure 3:**
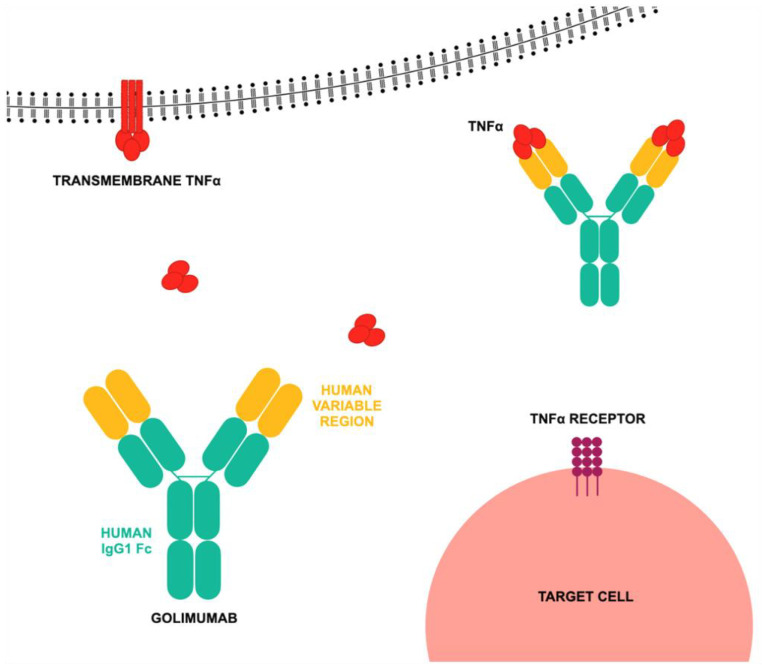
Mode of action of golimumab according to [65], where TNF-α—tumor necrosis factor alpha; IgG1 Fc—the crystallizable fragment of the immunoglobulin class G subclass 1. Golimumab exerts its effect by binding to TNF-α, preventing its interaction with TNF-α receptors on target cells. It is a monoclonal antibody with human-derived variable regions and a human IgG1 Fc fragment. Golimumab exhibits high affinity and specificity for both the soluble and membrane-bound forms of TNF-α, effectively neutralizing their activity by blocking their interaction with TNF-α cell surface receptors. Golimumab’s binding to human TNF-α inhibits the TNF-α-induced expression of adhesion molecules, including E-selectin, vascular cell adhesion molecule (VCAM), and intercellular adhesion molecule (ICAM), on human endothelial cells.

Golimumab’s fully human monoclonal antibody structure enables it to neutralize TNF-α in both soluble and membrane-bound forms, preventing receptor interaction [66,67,68,69]. The mechanism of action of golimumab is presented in Figure 3. Although structurally similar to infliximab, golimumab’s humanized design may elicit a different immune response. Developed via recombinant DNA technology, golimumab has been evaluated in large-scale, randomized, placebo-controlled Phase III trials, confirming its efficacy in reducing RA symptoms. Approved by the FDA in 2009 as Simponi, it is administered in Europe as a 50 mg subcutaneous injection monthly, either alone or with methotrexate (MTX). It is also indicated for PsA, axial spondyloarthritis (AxSpA), and polyarticular juvenile idiopathic arthritis (pJIA) in children over 40 kg. In cases of inadequate response, the dosage may be increased, though with higher risk of severe adverse effects. For ulcerative colitis, an initial 200 mg dose is followed by 100 mg in week two and then 50 mg or 100 mg every four weeks depending on patient response and weight. Golimumab effectively reduces inflammation, prevents cartilage degradation, and limits bone destruction. Pharmacokinetic studies indicate dose proportionality, peak plasma levels within 2–7 days post-injection, and steady-state concentrations achieved after 12 weeks. Its bioavailability is approximately 50%, with a half-life of about 12 days. Co-administration with MTX increases steady-state concentrations due to reduced clearance [70,71,72,73,74,75,76,77,78]. In newly diagnosed children and young adults with overt type 1 diabetes, golimumab improved endogenous insulin production and reduced the need for exogenous insulin compared with placebo [79]. Clinical trials have shown that FDA-approved golimumab doses, when combined with MTX, are significantly more effective than placebo in treating active RA. Short-term studies indicate a favorable safety profile with no significant increase in serious infections, malignancies, tuberculosis, or mortality [80,81,82]. The National Institute for Health and Care Excellence (NICE) found golimumab to be cost-effective compared with some approved alternatives, though not the most cost-effective option [83]. Clinical studies confirm golimumab’s efficacy in achieving ACR20/50/70 response rates, lowering DAS28 scores, and effectively treating AS and PsA. Its once-monthly subcutaneous administration allows for self-injection. Adverse effects are comparable to other biologics, but golimumab has lower discontinuation rates due to side effects compared with infliximab and a reduced risk of serious infections relative to certolizumab pegol. However, further research is necessary to evaluate long-term effects [84]. While anti-TNF-α therapies have enabled clinical remission in RA patients, the goal of achieving drug-free remission remains unmet [85]. In real-world practice, transitioning to golimumab has been effective in Japanese patients who had an inadequate response to first-line biologic treatments, regardless of whether their initial biologic was a TNF inhibitor or a non-TNF inhibitor. IV golimumab’s safety profile is consistent across rheumatologic conditions and comparable to other TNF inhibitors, including subcutaneous golimumab. However, concomitant use of MTX or corticosteroids has been linked to higher incidence of specific adverse events [20,86]. Recent research indicates that CDAI score changes in the golimumab-only group are similar to those in the golimumab + MTX group over one year, regardless of prior biologic use, with a consistent adverse event profile in both groups [87]. Additionally, in elderly RA patients with renal dysfunction, golimumab treatment continuation and adverse event occurrence were not affected by initial renal function. However, MTX use and low baseline CRP levels influenced treatment persistence [88]. Long-term evidence supports golimumab’s effectiveness for chronic immune-mediated rheumatic diseases, whether used as a first-line therapy for RA, PsA, and AS or as a second-line option for RA [89]. Another study reinforced previous findings, confirming the effectiveness and long-term use of golimumab as a second-line anti-TNF treatment for RA, PsA, and AxSpA, extending observations from six months to one year [90].

**Figure 4:**
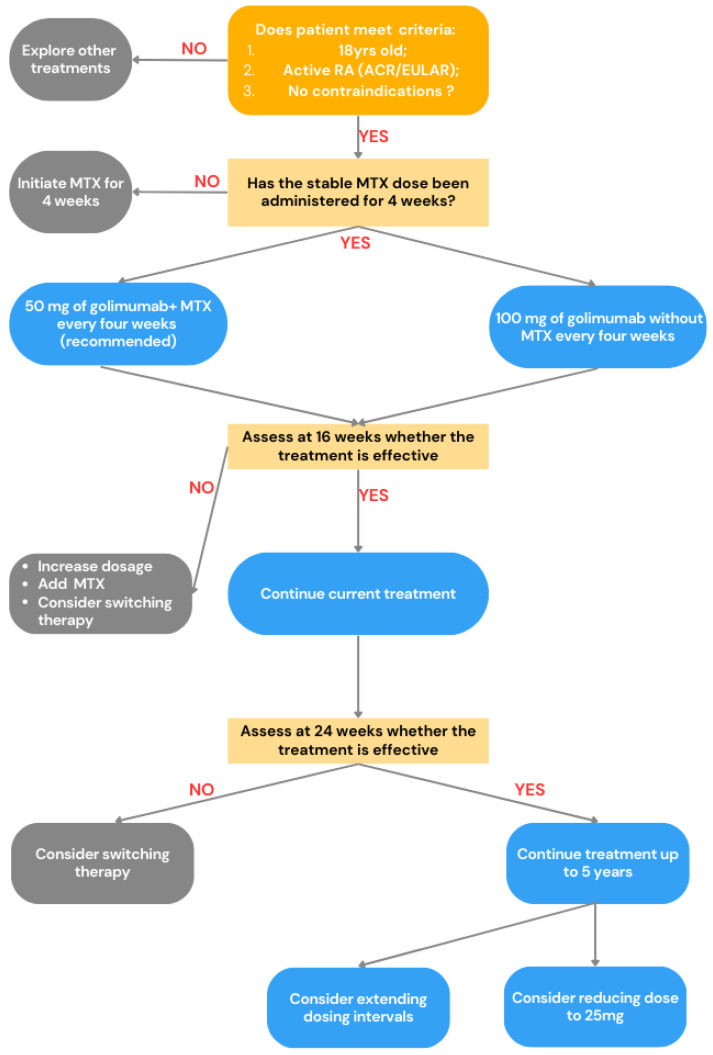
Brief summary of golimumab therapy pathway for rheumatoid arthritis (RA) according to [91], where RA—rheumatoid arthritis; ACR—American College of Rheumatology; EULAR—European League Against Rheumatism; MTX—methotrexate.

Golimumab is prescribed for adult patients with active rheumatoid arthritis who have shown an inadequate response to methotrexate treatment. To qualify, patients must have at least four swollen and tender joints, along with additional indicators such as elevated C-reactive protein (CRP) or erythrocyte sedimentation rate (ESR), morning stiffness, detectable bone erosion on imaging, or the presence of anti-citrullinated protein antibodies (ACPA) or rheumatoid factor (RF). Before initiating therapy, patients should have been on a stable methotrexate dose of 15 to 25 mg per week for a minimum of four weeks. Golimumab is contraindicated in individuals with hypersensitivity to the drug, prior use of TNF-α inhibitors, active or recurrent infections, a history of malignancies, or concurrent treatment with other immunosuppressive agents [91]. The recommended regimen consists of a 50 mg subcutaneous injection every four weeks in combination with methotrexate, though in certain cases, a 100 mg dose may be administered without MTX. At 16 weeks, treatment response is evaluated, and if the number of swollen and tender joints has improved by less than 20%, the dose may be increased to 100 mg every four weeks, or methotrexate may be introduced. A final assessment occurs at 24 weeks, and if clinical benefits persist, therapy may continue for up to five years. In real-world settings, dose adjustments are possible for patients who achieve sustained remission, either by extending the dosing interval or lowering the dosage. Conversely, for those with persistent disease activity, the dose may be escalated to 100 mg [91,92]. To visualize the decision-making algorithm for the use of golimumab in RA treatment, we created a graphical summary in Figure 4.

### 2.3. IL-6R Inhibitor—Sarilumab

**Figure 5:**
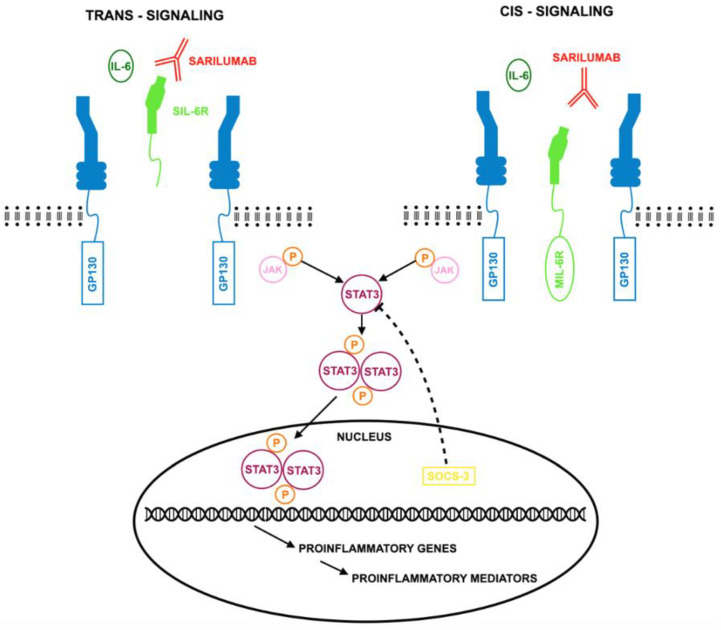
Mode of action of sarilumab according to [98], where IL-6—interleukin-6; MIL-6R—membrane-bound interleukin-6 receptor; SIL-6R—soluble interleukin-6 receptor; GP130—transmembrane glycol protein 130; JAK—Janus family tyrosine kinases; STAT—signal transducers and activators of transcription; SOCS-3—suppressors of cytokine signaling; P—phosphoryl group. Sarilumab selectively and strongly binds to a distinct site on IL-6R, including both its membrane-bound and soluble forms, effectively inhibiting both cis- and transactivation of IL-6 signaling. IL-6 interacts with its receptor and functions through both cis- and trans-signaling pathways. The fully functional IL-6 receptor consists of membrane-bound IL-6 receptor-α, which is associated with IL-6, and the signal-transducing GP130 molecule. In the classical (cis) signaling pathway, IL-6 attaches to MIL-6R, leading to the activation of GP130. This activation triggers the phosphorylation of JAK1, JAK2, and TYK2 (tyrosine kinase 2), which subsequently stimulate STAT1 and STAT3, ultimately driving the transcription of pro-inflammatory molecules. In the trans-signaling pathway, IL-6 binds to soluble IL-6R, forming a complex that can activate any cell expressing GP130. Under normal physiological conditions, a soluble form of GP130 acts as a regulator, preventing IL-6 trans-signaling while allowing cis-signaling to proceed.

In recent years, the advent of biotechnological therapies has greatly transformed the treatment and disease progression of rheumatoid arthritis (RA). Interleukin-6 (IL-6) has been identified as a critical cytokine in RA pathogenesis, disrupting both innate and adaptive immunity and promoting the production of acute-phase proteins responsible for systemic disease manifestations. IL-6 plays a multifaceted role in the immune system, influencing B lymphocyte differentiation, inducing C-reactive protein (CRP) release from the liver, increasing vascular permeability (leading to joint swelling), and stimulating osteoclast formation via receptor activator of nuclear factor kappa-B ligand (RANKL), contributing to bone resorption [93]. IL-6 is part of a cytokine family including IL-6, IL-11, IL-27, IL-31, and others, all signaling through the IL-6 receptor (gp130). It is produced primarily by macrophages in response to infections or inflammation, playing a protective role by eliminating pathogens and aiding tissue repair. IL-6 is essential to both innate and adaptive immunity, with increased production at sites of inflammation by various immune cells, including monocytes, T lymphocytes, fibroblasts, and endothelial cells [94]. Tocilizumab, the first approved IL-6 receptor (IL-6R) inhibitor for RA, has paved the way for the development of other biologics targeting the IL-6 pathway. These include drugs targeting IL-6 itself (such as sirukumab, olokizumab, and clazakizumab) or blocking the IL-6 receptor (such as sarilumab). The mechanism of action of sarilumab is presented in Figure 5.

Clinical trials, including Phase II and Phase III studies, have shown that sarilumab is effective across various RA patient groups, including those with an inadequate response to methotrexate (MTX) and those who failed TNF inhibitors. Sarilumab monotherapy has demonstrated superior efficacy over adalimumab in MTX-intolerant patients. Although sarilumab has a similar safety profile to tocilizumab, it has higher binding affinity and a longer half-life, allowing for less frequent dosing (every two weeks instead of weekly). These characteristics contribute to the potential optimal placement of sarilumab in RA treatment. Real-world studies will provide further insights into its long-term effectiveness [95,96,97,98,99,100,101]. IL-6 is involved in multiple physiological processes, including neuroendocrine function, lipid metabolism, vascular disease, and neuropsychological behavior. It also plays a role in RA by facilitating communication between the immune system and the central nervous system. Three Phase III trials suggest that sarilumab may also address pain, mood disorders, and fatigue in RA patients [102]. Furthermore, IL-6 inhibitors, including sarilumab, have been explored as treatments for the hyperinflammatory phase of COVID-19, showing potential to improve patient outcomes in severe cases [103,104,105,106,107]. Therapeutic options for RA have expanded significantly over the past three decades, with various medications available. IL-6 inhibition, particularly in patients who cannot tolerate or respond to MTX, offers a promising strategy. Treat-to-profile approaches suggest that IL-6 inhibitors can effectively manage both articular and extra-articular RA symptoms, even in the presence of comorbidities or elevated inflammatory markers [108]. Sarilumab (Kevzara), a monoclonal antibody targeting IL-6R, is approved for moderate-to-severe RA in adults with an inadequate response or intolerance to DMARDs. Administered subcutaneously every two weeks, sarilumab reduces RA symptoms, improves physical function, and enhances health-related quality of life (HRQOL). When used with MTX, it slows structural damage progression, and as monotherapy, it is superior to adalimumab in patients who cannot tolerate MTX. Its safety profile is consistent with IL-6 inhibition, and anti-drug antibodies (ADAs) detected in a small subset of patients did not affect efficacy or safety. Overall, sarilumab provides a valuable treatment option for RA patients who have not adequately responded to or cannot tolerate at least one DMARD. Common side effects include injection site reactions, neutropenia, elevated liver enzymes, and serum cholesterol [93,109,110,111,112]. Sarilumab monotherapy has shown comparable efficacy to its combination with MTX [113]. However, non-medical switching from tocilizumab to sarilumab has not been shown to be non-inferior and may increase disease activity and reduce sarilumab persistence [114]. Sarilumab at 200 mg every two weeks also demonstrates greater inhibition of IL-6/STAT3 signaling compared with SC tocilizumab every two weeks, though with weaker inhibition than SC tocilizumab weekly [115]. In patients with inadequate responses to conventional DMARDs, 200 mg sarilumab monotherapy provides greater efficacy with a comparable safety profile to csDMARDs, adalimumab, and other biologic DMARDs [116]. Sarilumab, a strong IL-6 receptor antagonist, shows efficacy in improving various RA-related outcomes, including HAQ-DI, SF-36, and FACIT-F, across all RA subgroups. Monotherapy with sarilumab has demonstrated superior benefits over adalimumab, suggesting that IL-6 inhibition may be more effective than TNF blockade in managing RA [117]. In the UK, the Appraisal Committee deemed sarilumab cost-effective in certain RA patient groups, excluding those with TNFi-IR who are eligible for rituximab or those with moderate RA and a DAS28 score above 4.0 [118]. Sarilumab exposure is not significantly influenced by body weight, and no dose adjustments are needed based on this or other patient characteristics [119]. In patients with inadequate responses to csDMARDs or TNF inhibitors, sarilumab has demonstrated greater efficacy with comparable safety [120]. In Japanese RA patients with an inadequate response to MTX, sarilumab (at 150 mg or 200 mg every two weeks) showed sustained clinical efficacy, with safety profiles consistent with prior research [121]. The analysis of patient-reported outcomes (PROs) in clinical trials suggests that sarilumab improves RA-related quality of life [122]. A comparison of sarilumab and tocilizumab showed no significant differences in adverse events or laboratory changes, and the transition from IV to SC therapy did not introduce new safety concerns, with clinical effectiveness maintained over 96 weeks [123,124]. Open-label studies have shown that ADAs in sarilumab-treated patients do not significantly impact safety or efficacy over 24 weeks [125]. Long-term safety profiles remain consistent, and sarilumab also demonstrates effectiveness in patients with inadequate responses to JAK inhibitors and tocilizumab [126,127]. Sarilumab has been shown to reduce RA activity when combined with salazosulfapyridine and prednisolone without triggering a relapse of lymphoproliferative disorders (LPD). For patients with a history of LPDs, rituximab is preferred over other biologic DMARDs according to the American College of Rheumatology (ACR) [128,129]. Additionally, IL-6R gene polymorphisms, particularly rs4845625, could serve as biomarkers for predicting the efficacy and adverse responses to sarilumab in RA patients [130]. Reports also link sarilumab treatment to rheumatoid neutrophilic dermatosis (RND) in RA patients [131], but extended use has not raised new safety concerns [132]. Data from ARTIS show that b-/tsDMARDs used in RA treatment have generally acceptable safety profiles, with differences in tolerability and infection risks [133]. Sarilumab has shown effectiveness in maintaining sustained remission and reducing glucocorticoid doses in patients with polymyalgia rheumatica [134]. A systematic review suggests that IL-6R inhibitors, including sarilumab, may be effective alternatives to TNF blockers, particularly for patients not responding to tocilizumab [135].

**Figure 6:**
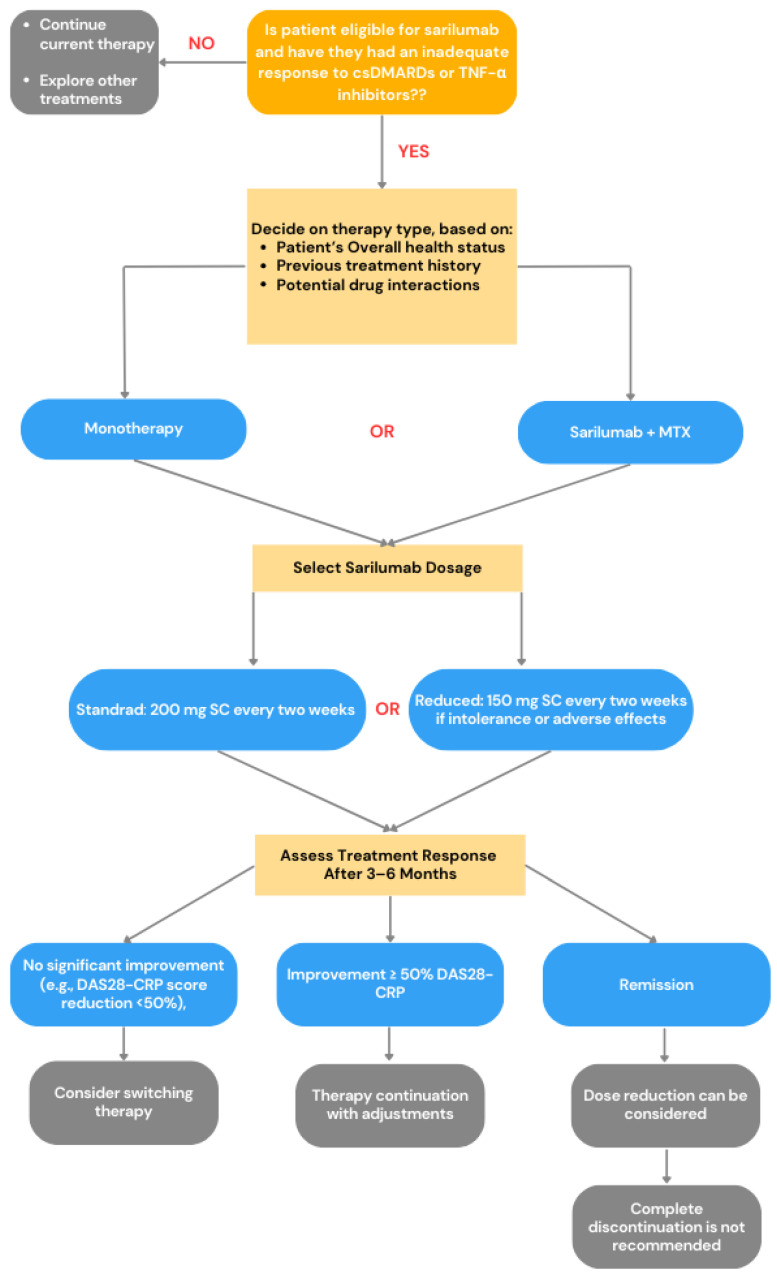
Brief summary of sarilumab therapy pathway for rheumatoid arthritis (RA) according to [14], where csDMARDs—conventional synthetic disease-modifying antirheumatic drugs; TNF-α—tumor necrosis factor alpha; MTX—methotrexate; SC—subcutaneous; DAS28-CRP—Disease Activity Score in 28 joints based on C-reactive protein level.

Sarilumab is prescribed for adults with moderate-to-severe rheumatoid arthritis who have not responded adequately to or cannot tolerate at least one csDMARD or TNF-alpha inhibitor [14]. It can be administered alone or in combination with other csDMARDs, particularly methotrexate. However, it is not suitable for patients with severe active infections, significant liver impairment, neutropenia (ANC below 500 cells/mm^3^), thrombocytopenia (platelet count under 50,000 cells/mm^3^), or hypersensitivity to any of its components. The recommended dosage is 200 mg subcutaneously every two weeks (Q2W). If adverse effects such as neutropenia or elevated liver enzymes occur, the dose may be lowered to 150 mg subcutaneously Q2W. Treatment effectiveness is evaluated between 3 and 6 months, and if there is no meaningful improvement (at least a 50% reduction in disease activity based on the DAS28-CRP scale), switching to an alternative therapy is advised. In cases where remission is achieved, dose reduction may be considered, but full discontinuation is not recommended. Routine monitoring is necessary, including blood count tests every 4–8 weeks initially, followed by every 12 weeks, to detect potential neutropenia or thrombocytopenia. Liver enzyme levels (ALT and AST) and lipid profiles should also be monitored, as treatment may lead to increased cholesterol and triglyceride levels. The most frequently reported side effects include upper respiratory infections, neutropenia (19.4 cases per 100 patient-years), injection site reactions (42.4 cases per 100 patient-years), and leukopenia (23.7 cases per 100 patient-years). Serious infections, such as sepsis and opportunistic infections, occur at a rate of 3.3 per 100 patient-years [14,124]. Long-term studies have not identified any new safety concerns related to sarilumab. Overall, it remains an effective treatment option for RA patients who have not benefited from other therapies. However, the ongoing monitoring of blood counts and liver function is essential to ensuring safe use. To visualize the decision-making algorithm for the use of Sarilumab in RA treatment, we created a graphical summary in Figure 6.

## 3. Conclusions

The introduction of disease-modifying antirheumatic drugs (DMARDs) has revolutionized arthritis management. Conventional synthetic DMARDs (csDMARDs) such as methotrexate have long been the cornerstone of treatment, but their limitations in achieving sustained remission have necessitated the development of biologic (boDMARDs and bsDMARDs) and targeted synthetic (tsDMARDs) DMARDs. Innovations such as abatacept, golimumab, and sarilumab have provided new therapeutic options targeting specific immune mechanisms, enhancing patient outcomes, particularly in those with refractory disease. Abatacept, a CTLA4-Ig fusion protein, has demonstrated efficacy in modulating T-cell activation, making it a valuable option for RA patients unresponsive to traditional therapies. Similarly, golimumab, a TNF inhibitor, offers effective disease control and structural preservation, with a favorable safety profile compared with some other anti-TNF agents. Meanwhile, sarilumab, an IL-6 receptor antagonist, has emerged as a potent alternative, particularly for patients intolerant to methotrexate or other TNF inhibitors. Its impact extends beyond joint inflammation, addressing systemic symptoms such as fatigue and cardiovascular risks associated with RA. While biologic and targeted therapies have significantly improved disease management, challenges remain, including treatment costs, accessibility, and patient response variability. Biosimilars have played a crucial role in increasing access to biologic therapies, though their long-term efficacy and immunogenicity require continued evaluation. Future research should focus on personalized medicine approaches to better predict treatment responses, minimize adverse effects, and optimize long-term disease control. Advances in biomarkers, pharmacogenomics, and precision immunology will likely refine RA treatment strategies, leading to more individualized and effective care. Additionally, exploring novel therapeutic targets and combination strategies may further enhance patient outcomes and achieve the ultimate goal of drug-free remission.

## Figures and Tables

**Table 1:** Brief summary of ACR/EULAR classification criteria for RA according to [13]. ACR—American College of Rheumatology; EULAR—European League Against Rheumatism; RA—rheumatoid arthritis; RF—rheumatoid factor; ACPAs—anti-citrullinated protein antibodies; CRP—C-reactive protein; ESR—erythrocyte sedimentation rate.

Criterion	Category	Points
Joint involvement	1 large joint	0
	2–10 large joints	1
	1–3 small joints (with or without large joints)	2
	4–10 small joints (with or without large joints)	3
	More than 10 joints (including at least 1 small joint)	5
Serology (RF or ACPAs)	Negative	0
	Low-positive	2
	High-positive	3
Acute-phase reactants (CRP or ESR)	Normal	0
	Abnormal	1
Symptom duration	Less than 6 weeks	0
	6 weeks or more	1

**Table 2:** Summary table: RA management algorithm according to [15]. ACR: American College of Rheumatology; EULAR: European League Against Rheumatism; RA: rheumatoid arthritis; MTX: methotrexate; DAS28: Disease Activity Score 28; CDAI: Clinical Disease Activity Index; bDMARD: biologic disease-modifying antirheumatic drug; tsDMARD: targeted synthetic disease-modifying antirheumatic drug; JAK: JAK-kinase inhibitor; DMARD: disease-modifying antirheumatic drug.

Step	Action	Considerations
1. Diagnosis	Confirm RA by using ACR/EULAR criteria	Assess joint involvement, serology, and inflammatory markers
2. Initial treatment	Start MTX ± short-term glucocorticoids	If MTX is contraindicated, use leflunomide or sulfasalazine
3. Monitoring (3–6 months)	Assess DAS28 or CDAI	If no improvement at 3 months, adjust treatment
4. Escalation	Add bDMARD or tsDMARD in high-risk patients	Consider JAK inhibitors with caution
5. Failure of advanced therapy	Switch to another bDMARD or tsDMARD	Choose a drug with a different mechanism of action
6. Remission and Tapering	Reduce DMARD dose gradually	Complete discontinuation is not recommended

**Table 3:** Disease Activity Score-28 according to [15].

Disease Activity Level	DAS28 Score Range	Mean DAS28 ± SD
Remission	<2.6	1.99 ± 0.38
Low Disease Activity	2.6–3.2	3.04 ± 0.17
Moderate Disease Activity	3.2–5.1	4.25 ± 0.58
High Disease Activity	>5.1	6.38 ± 0.87

**Table 4:** Clinical Disease Activity Index according to [15].

Disease Activity Level	CDAI Score Range	Mean CDAI ± SD
Remission	<2.8	0.90 ± 0.65
Low Disease Activity	2.8–10	6.45 ± 2.35
Moderate Disease Activity	10–22	16.46 ± 3.31
High Disease Activity	>22	38.56 ± 11.88

**Table 5:** The ACR improvement criteria for RA according to [13]. ACR: American College of Rheumatology; RA: rheumatoid arthritis; TJC: Tender Joint Count; SJC: Swollen Joint Count.

Criterion Category	Parameters	Score
ACR20	≥20% improvement in Tender Joint Count (TJC) and Swollen Joint Count (SJC), plus ≥20% improvement in at least 3 of the 5 additional indicators listed above	≥20% improvement
ACR50	≥50% improvement in TJC and SJC, plus ≥50% improvement in at least 3 of the 5 additional indicators listed above	≥50% improvement
ACR70	≥70% improvement in TJC and SJC, plus ≥70% improvement in at least 3 of the 5 additional indicators listed above	≥70% improvement

## Data Availability

Data sharing is not applicable to this article.
